# Supplementary material for: Structural insights into the competitive inhibition of the ATP-gated P2X receptor channel
Source: Nat Commun. 2017 Oct 12;8:876. doi: 10.1038/s41467-017-00887-9 (PMC5638823; doi:10.1038/s41467-017-00887-9)
Supplement: Supplementary file 1 — Supplementary Information [file 41467_2017_887_MOESM1_ESM.pdf]

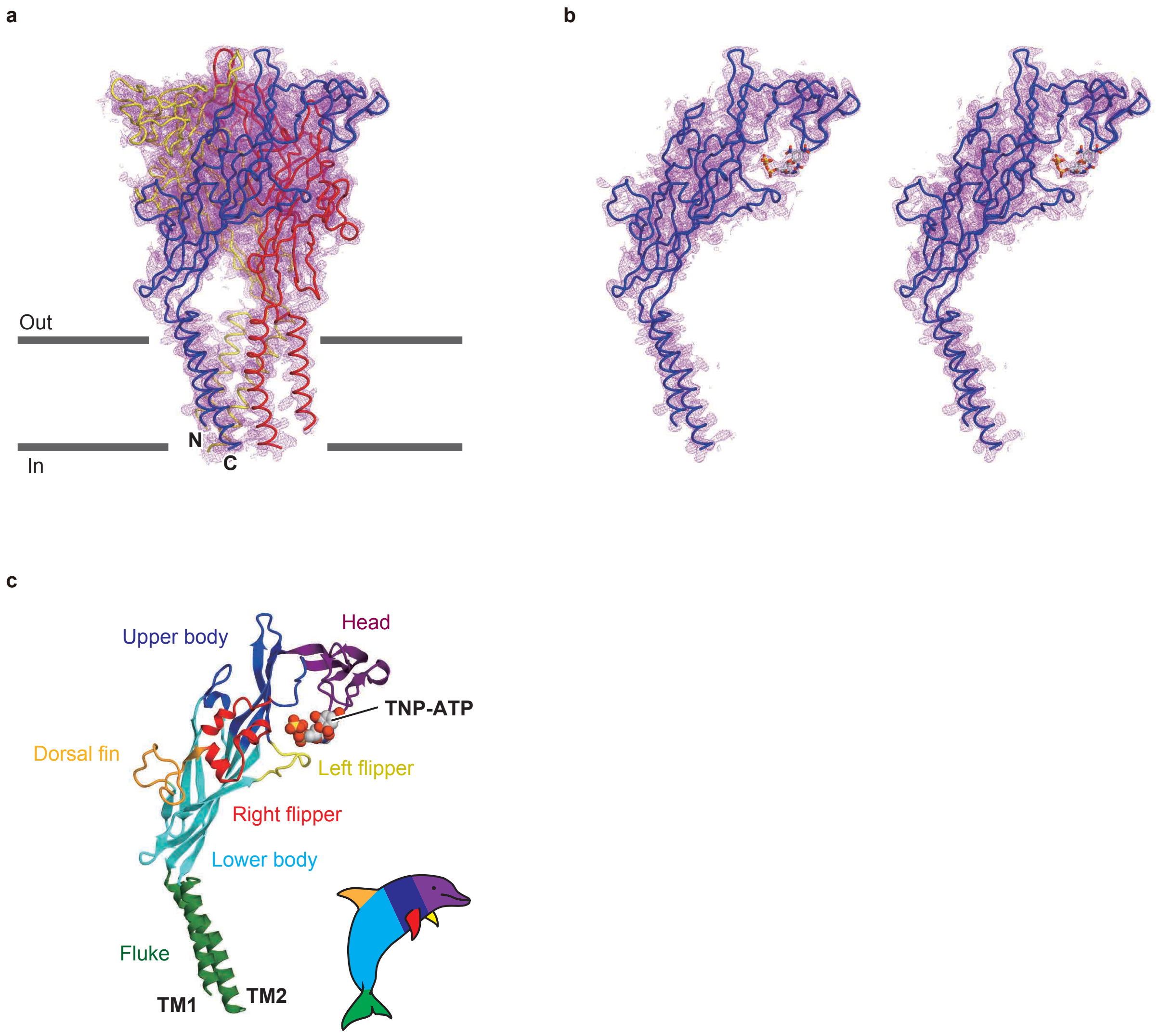

Supplementary Figure 1 The electron density map and the subunit fold of ckP2X7$_{cryst}$.

(**a**) The $2F_o$ - F_c density map of ckP2X7$_{cryst}$, contoured at 1 σ. (**b**) The stereo view showing $2F_o$ - F_c density map of the ckP2X7$_{cryst}$ subunit, contoured at 1 σ. (**c**) Subunit of the TNP-ATP-bound ckP2X7$_{cryst}$, colored according to the previously proposed dolphin-like model[1]. Based on the sequence alignments, we defined the Fluke (Cys28 - Lys49, and Ser316 - Asn346), Lower body (Arg50 - Val68, Thr81 - Phe91, Phe176 - Asn195, Gly238 - Arg265, and Arg303 - Phe315), Upper body (Ala69 - Tyr80, Val92 - Asn101, Pro157 - Pro164, and Phe277 - Ile302), Head (Gln102 - Cys156), Right flipper (Pro165 - Asp175, and Leu219 - Gly237), Left flipper (Leu266 - Gly276), and Dorsal fin (Ile196 - Arg218) domains of the ckP2X7$_{cryst}$.

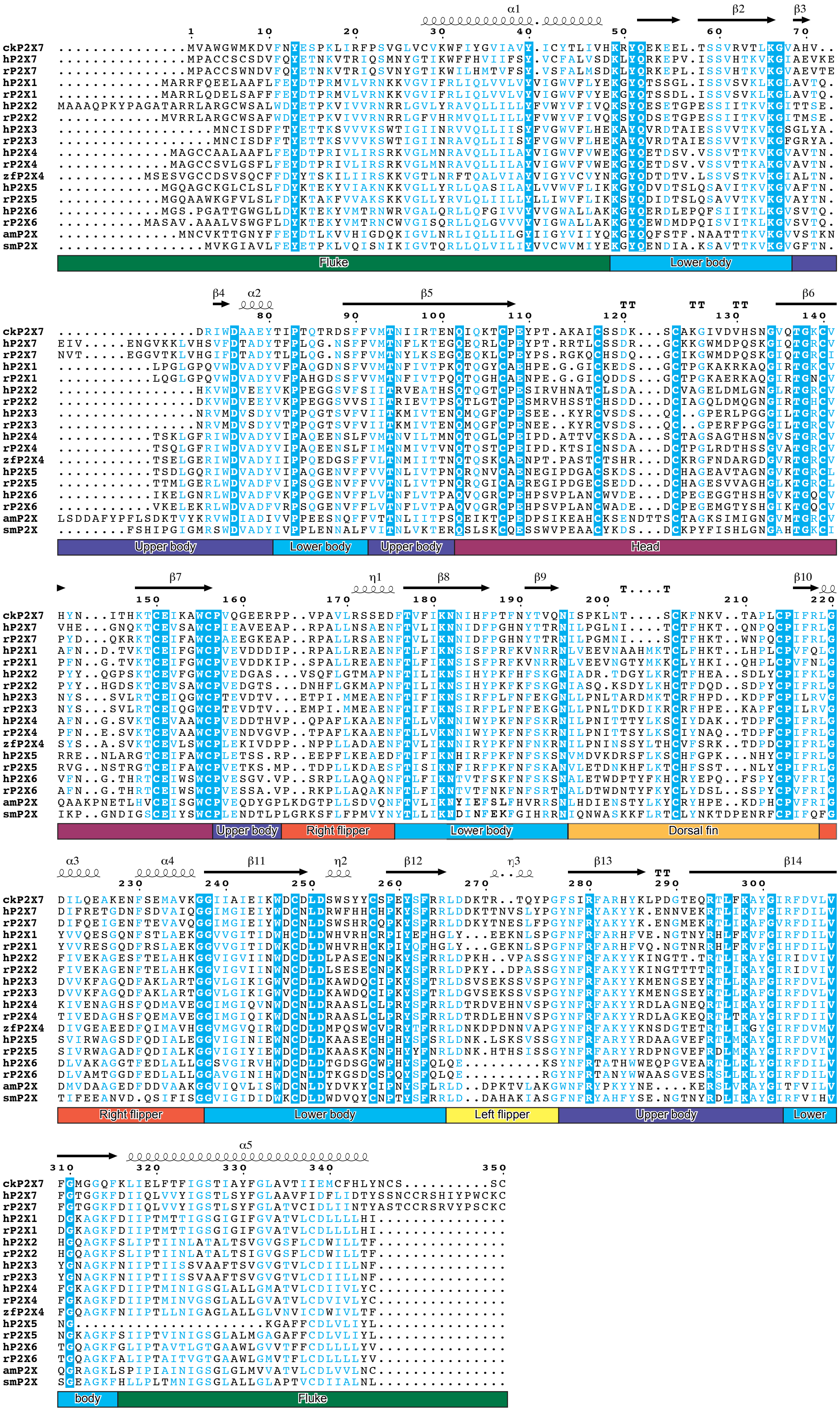

ckP2X7
hP2X7
rP2X7
hP2X1
rP2X1
hP2X2
rP2X2
hP2X3
rP2X3
hP2X4
rP2X4
zfP2X4
hP2X5
rP2X5
hP2X6
rP2X6
amP2X
smP2X
α1
β2
β3
β4
α2
β5
β6
β7
η1
β8
β9
β10
α3
α4
β11
η2
β12
η3
β13
β14
α5
Fluke
Lower body
Upper body
Head
Right flipper
Dorsal fin
Left flipper

Supplementary Figure 2 Sequence alignment of P2X receptors.

The amino acid sequences of P2X receptors were aligned using Clustal Omega (https://www.ebi.ac.uk/Tools/msa/clustalo/), and are shown using ESPript3 (http://espript.ibcp.fr/ESPript/ESPript/). Secondary structure elements from the ckP2X7$_{cryst}$ are depicted in cartoon representations and are labeled above the alignments, and the colors of the domains based on the dolphin-like model are presented below the alignments. For the sequence alignment, the following vertebrate and invertebrate P2X receptors were used: chicken (ckP2X7, NCBI Accession Number: XP_001235163), human (hP2X1, NP_002549; hP2X2, NP_733782; hP2X3, NP_002550; hP2X4, Q99571; hP2X5, Q93086; hP2X6, AAF13303; and hP2X7, AAH11913), rat (rP2X1, P47824; rP2X2, NP_446108; rP2X3, CAA62594; rP2X4, AAC52380; rP2X5, CAA63052; rP2X6, CAA63053; and rP2X7, CAA65131), zebrafish (zfP2X4, AAK00945), Gulf Coast tick (amP2X, AEO34575), and blood fluke (smP2X, CAH04147).

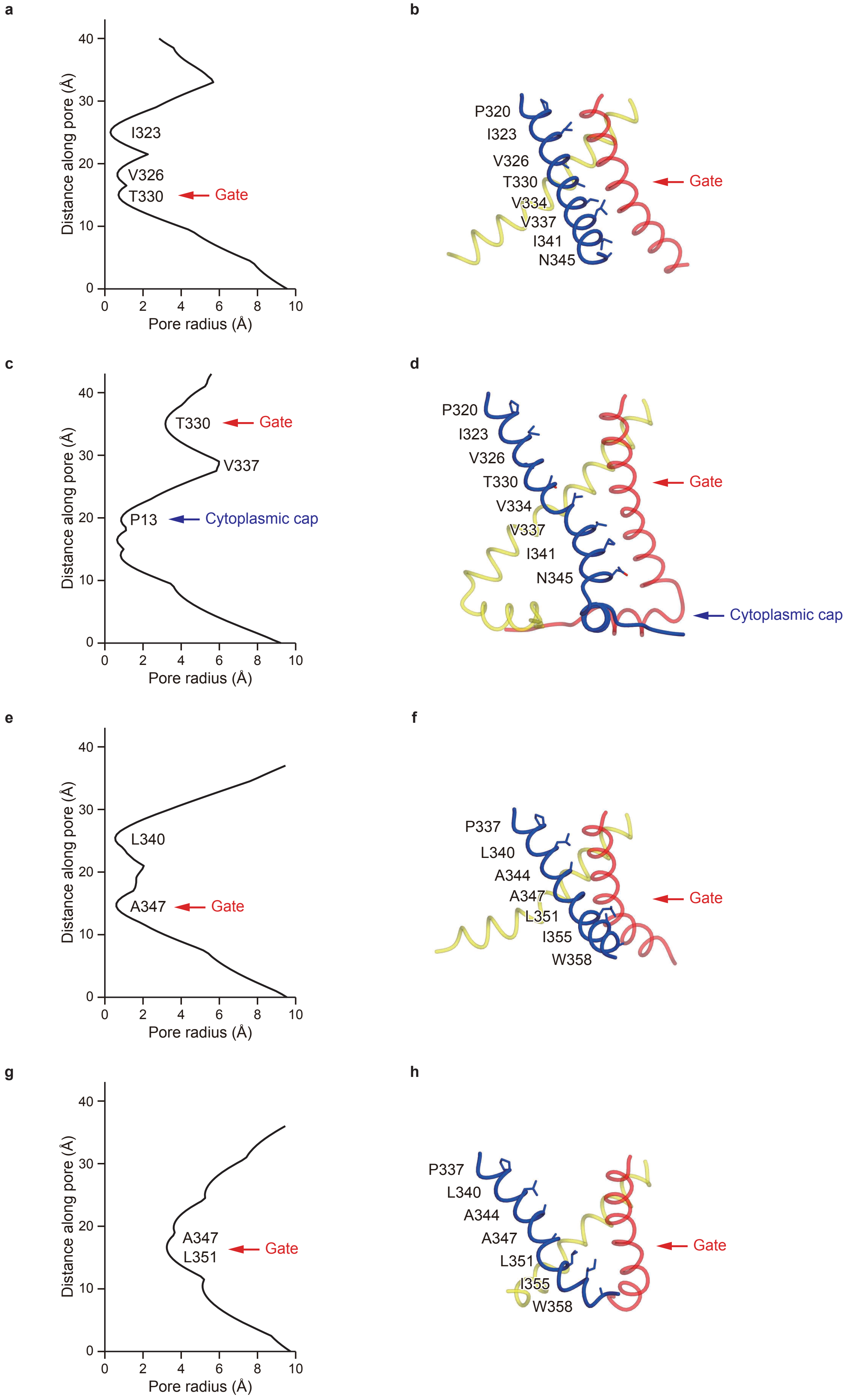
a
b
c
d
e
f
g
h
Distance along pore (Å)
Pore radius (Å)
0
2
4
6
8
10
20
30
40
I323
V326
T330
Gate
P320
V334
V337
I341
N345
V337
P13
Cytoplasmic cap
L340
A347
P337
A344
L351
I355
W358

Supplementary Figure 3 The transmembrane pores of the apo, closed and ATP-bound, open hP2X3 and zfP2X4 structures.

(**a**) The pore radius for the apo, closed hP2X3 structure (PDB ID: 5SVJ) along the pore center axis. (**b**) Pore-lining residues of the apo, closed hP2X3 structure are depicted by stick models. (**c**) The pore radius for the ATP-bound, open hP2X3 structure (PDB ID: 5SVK) along the pore center axis. (**d**) Pore-lining residues of the ATP-bound, open hP2X3 structure are depicted by stick models. (**e**) The pore radius for the apo, closed zfP2X4 structure (PDB ID: 4DW0) along the pore center axis. (**f**) Pore-lining residues of the apo, closed zfP2X4 structure are depicted by stick models. (**g**) The pore radius for the ATP-bound, open zfP2X4 structure (PDB ID: 4DW1) along the pore center axis. (**h**) Pore-lining residues of the ATP-bound, open zfP2X4 structure are depicted by stick models. The pore size was calculated by the program HOLE.

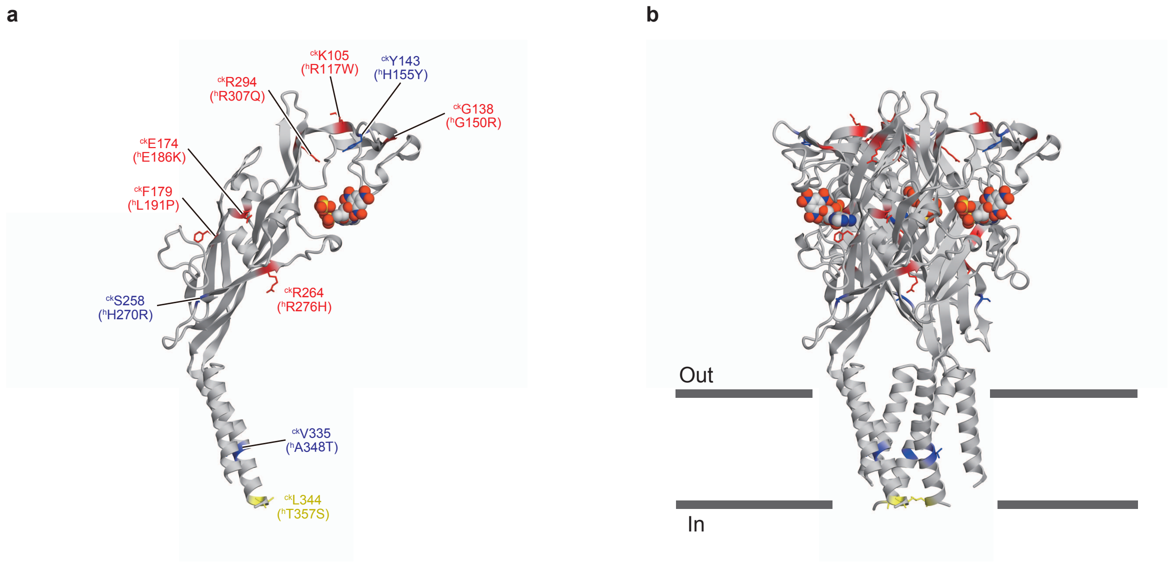

c

dsSNP ID	Amino acid change in hP2X7	Corresponding residue in ckP2X7	Effect on function	Related disease
rs17525809	^{h}V76A	-	Partial loss	Mood disorder
rs28360445	^{h}R117W	^{ck}K105	Loss	Mood disorder
rs28360447	^{h}G150R	^{ck}G138	Loss	Accelerated Lumbar Spine Bone Loss
rs208294	^{h}H155Y	^{ck}Y143	Gain	Crohn's disease, Papillary thyroid cancer, Bipolar or unipolar disorder
rs28360451	^{h}E186K	^{ck}E174	Loss	
rs28360452	^{h}L191P	^{ck}F179	Loss	Mood disorder
rs7958311	^{h}H270R	^{ck}S258	Gain	
rs7958316	^{h}R276H	^{ck}R264	Loss	
rs28360457	^{h}R307Q	^{ck}R294	Loss	Accelerated Lumbar Spine Bone Loss, Fracture risk, Periprosthetic osteolysis, Crohn's disease
rs1718119	^{h}A348T	^{ck}V335	Gain	Toxoplasmosis, Anxiety, Bipolar or unipolar disorder
rs2230911	^{h}T357S	^{ck}L344	Partial loss	Mood disorder

Supplementary Figure 4 The locations of amino acid residues substituted by SNPs in the hP2X7 receptor. (**a,b**) The amino acid residues in the hP2X7 receptor that are substituted by SNPs and involved in losses or gains of functions are mapped on the single subunit (**a**) or the trimer (**b**) of the ckP2X7$_{cryst}$ structure. The substituted amino acid residues are depicted by stick models. Red, yellow and blue colors indicate loss of function, partial loss of function and gain of function, respectively. (**c**) The relationship between the amino acid residues substituted by SNPs in the hP2X7 receptor and ckP2X7$_{cryst}$. The table was created based on Sluyter and Stokes, 2011 (ref. 2) and Roger *et al.*, 2010 (ref. 3). The superscripts "ck" and "h" refer to the chicken and human P2X7 receptors.

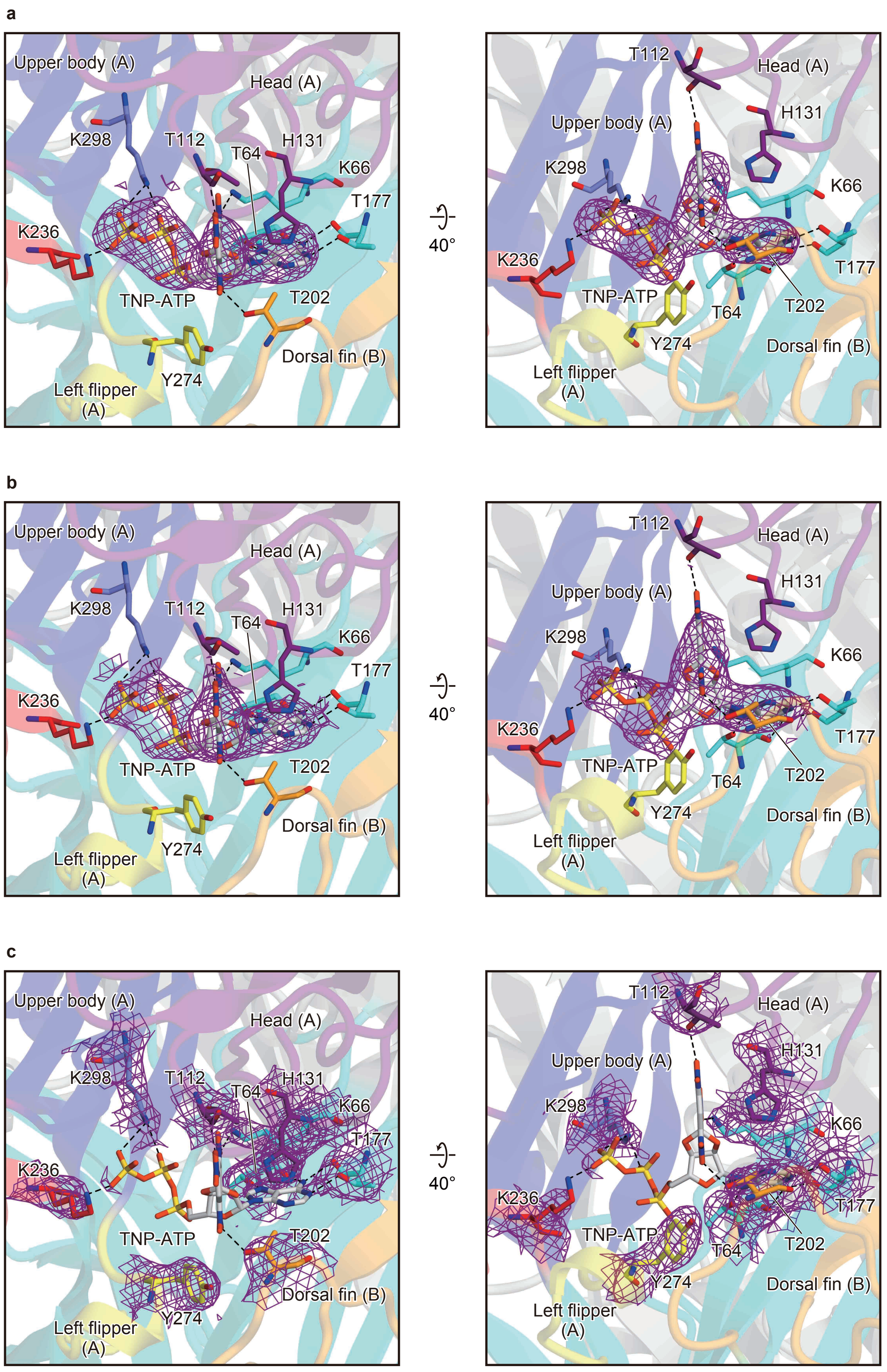

Supplementary Figure 5 Density of TNP-ATP and surrounding residues in the TNP-ATP binding site. (**a,b**) The F_o - F_c density map contoured at 2.5 σ (**a**) and the $2F_o$ - F_c density map contoured at 1 σ (**b**), showing the TNP-ATP. (**c**) The $2F_o$ - F_c density map contoured at 1 σ, showing the residues involved in TNP-ATP binding. The TNP-ATP molecule and the residues involved in TNP-ATP binding are depicted by stick models.

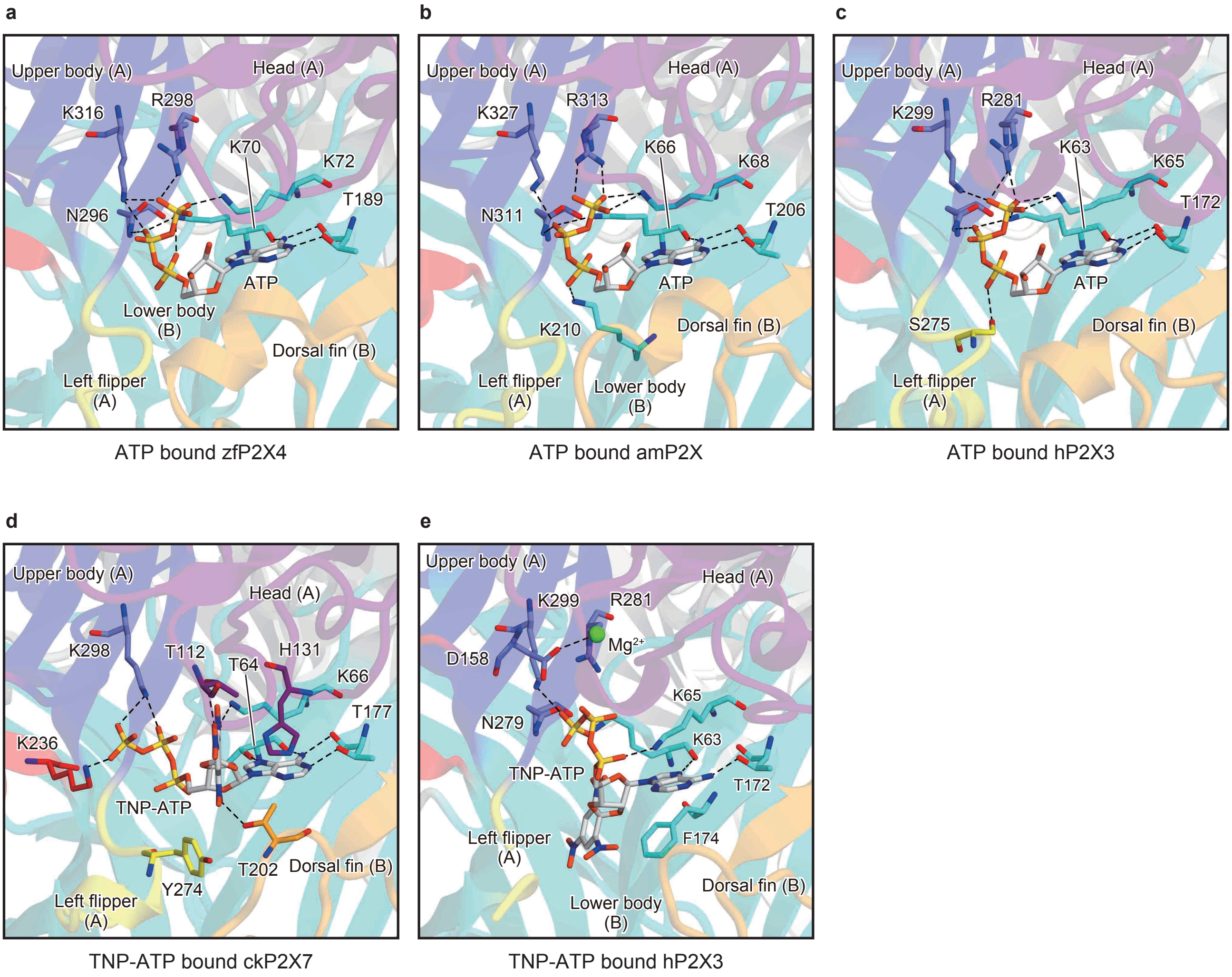

Supplementary Figure 6 Comparison of ATP and TNP-ATP binding sites.

(**a-c**) Close-up views of the ATP binding sites in the ATP-bound zfP2X4 structure (PDB ID: 4DW1) (**a**), the ATP-bound amP2X structure (PDB ID: 5F1C) (**b**) and the ATP-bound hP2X3 structure (PDB ID: 5SVK) (**c**). (**d,e**) Close-up views of the TNP-ATP binding site in the TNP-ATP-bound ckP2X7 structure (**d**) and the TNP-ATP-bound hP2X3 structure (PDB ID: 5SVQ) (**e**). The amino acid residues, ATP and TNP-ATP are depicted by stick models. The molecule is colored according to the dolphin-like model. Dotted black lines indicate hydrogen bonds (<3.3 Å).

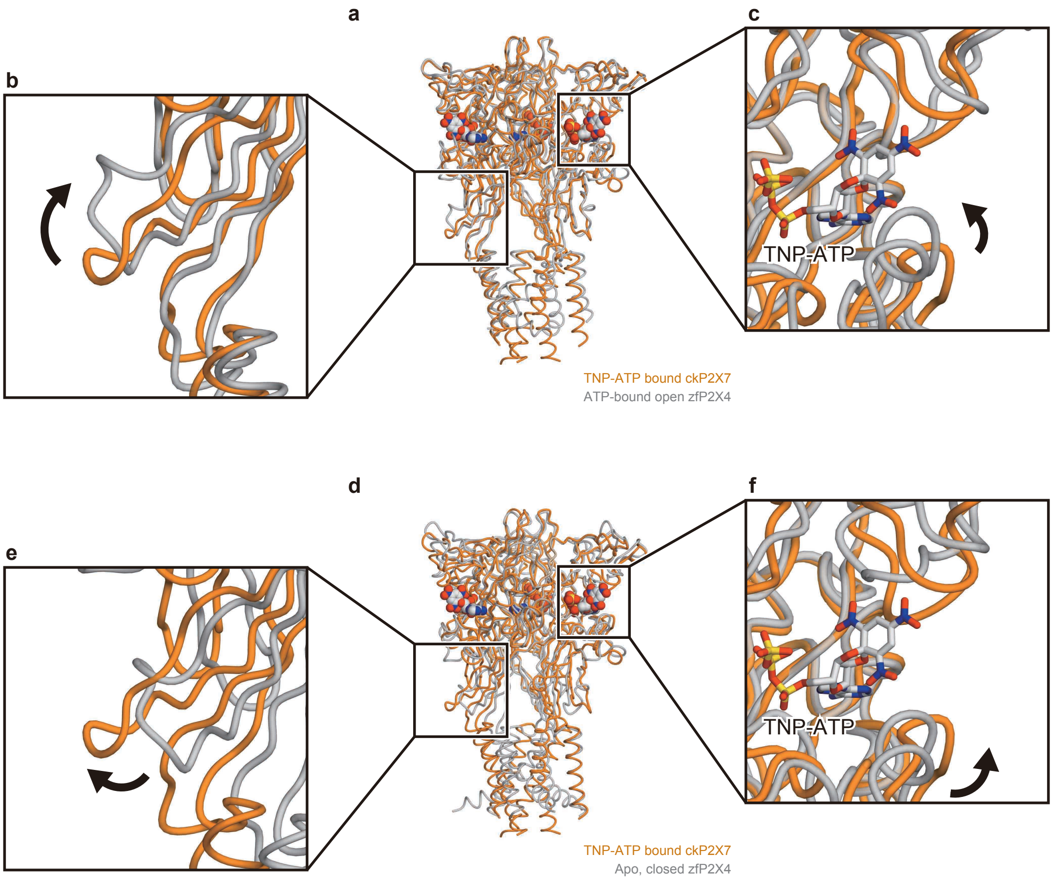

Supplementary Figure 7 Comparison of the overall structures of the apo closed, and TNP-ATP-bound and ATP-bound, open states for ckP2X7 and zfP2X4.

(**a-c**) Subunit comparisons of the TNP-ATP bound ckP2X7 (orange) and ATP-bound, open zfP2X4 (gray, PDB ID: 4DW1) structures. Close-up views of the lower body domain (**b**) and the TNP-ATP binding site in ckP2X7 (**c**) are shown in each box. The black arrows denote the movement from the TNP-ATP-bound state to the ATP-bound open state. (**d-f**) Subunit comparisons of the TNP-ATP-bound ckP2X7 (orange) and apo, closed zfP2X4 (gray, PDB ID: 4DW0) structures. Close-up views of the lower body domain (**e**) and the TNP-ATP binding site in ckP2X7 (**f**) are shown in each box. The black arrows denote the movement from the apo, closed state to the TNP-ATP-bound state.

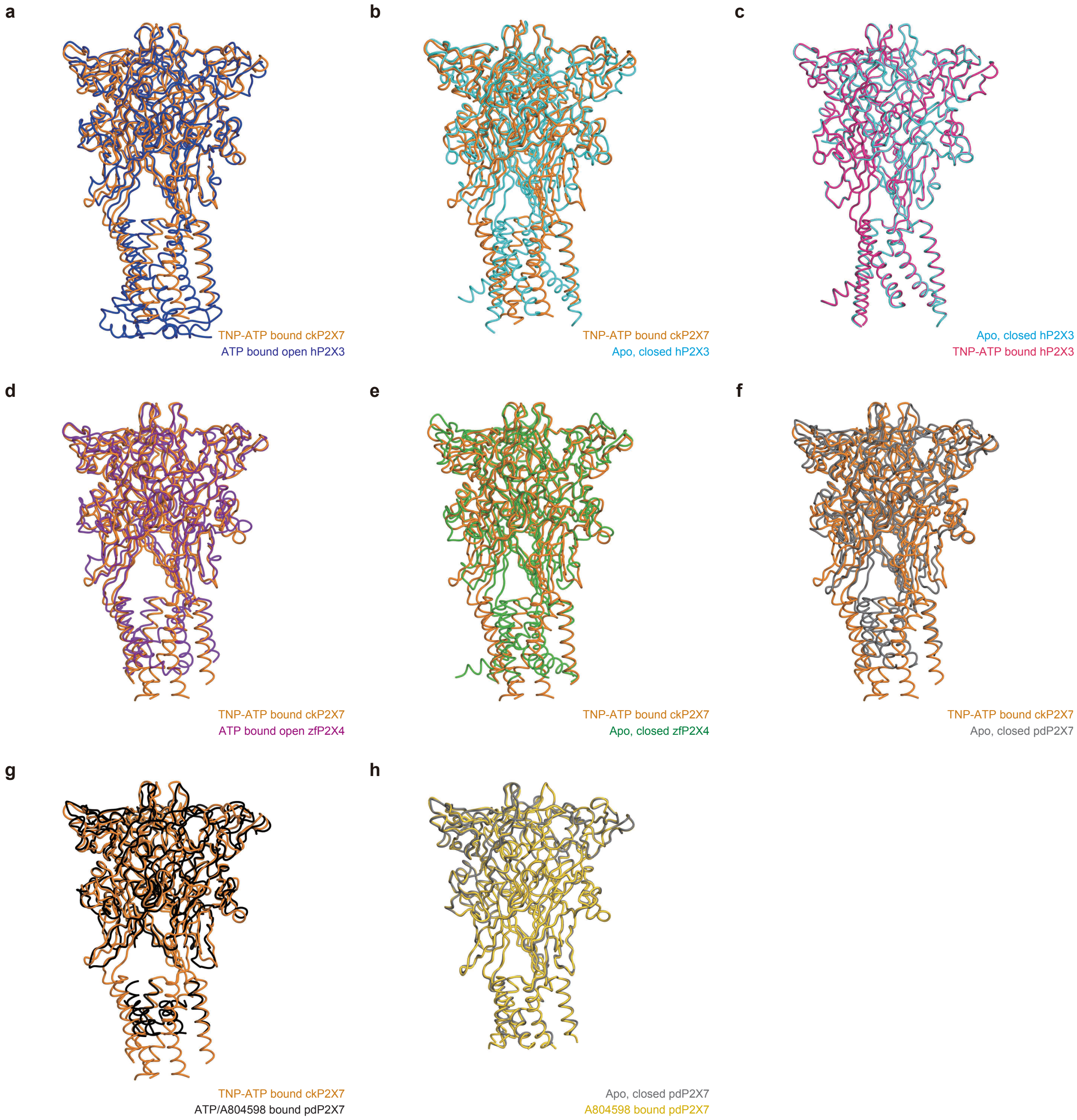

a
TNP-ATP bound ckP2X7
ATP bound open hP2X3
b
TNP-ATP bound ckP2X7
Apo, closed hP2X3
c
Apo, closed hP2X3
TNP-ATP bound hP2X3
d
TNP-ATP bound ckP2X7
ATP bound open zfP2X4
e
TNP-ATP bound ckP2X7
Apo, closed zfP2X4
f
TNP-ATP bound ckP2X7
Apo, closed pdP2X7
g
TNP-ATP bound ckP2X7
ATP/A804598 bound pdP2X7
h
Apo, closed pdP2X7
A804598 bound pdP2X7

Supplementary Figure 8 Comparison of the overall structures for ckP2X7, hP2X3, zfP2X4 and pdP2X7.

(**a**) The superimposition of the TNP-ATP-bound ckP2X7 (orange) and ATP-bound, open hP2X3 (blue, PDB ID: 5SVK) structures. The RMSD value is 1.87 Å for 856 Cα atoms between trimers. (**b**) The superimposition of the TNP-ATP-bound ckP2X7 (orange) and apo, closed hP2X3 (cyan, PDB ID: 5SVJ) structures. The RMSD value is 2.31 Å for 758 Cα atoms between trimers. (**c**) The superimposition of the apo, closed hP2X3 (cyan) and TNP-ATP-bound hP2X3 (pink, PDB ID: 5SVQ) structures. The RMSD value is 0.22 Å for 954 Cα atoms between trimers. (**d**) The superimposition of the TNP-ATP-bound ckP2X7 (orange) and ATP-bound, open zfP2X4 (purple, PDB ID: 4DW1) structures. The RMSD value is 1.95 Å for 836 Cα atoms between trimers. (**e**) The superimposition of the TNP-ATP-bound ckP2X7 (orange) and apo, closed zfP2X4 (green, PDB ID: 4DW0) structures. The RMSD value is 2.35 Å for 814 Cα atoms between trimers. (**f**) The superimposition of the TNP-ATP-bound ckP2X7 (orange) and ATP/antagonist (A804598) bound pdP2X7 (black, PDB ID: 5U2H) structures. The RMSD value is 1.73 Å for 790 Cα atoms between trimers. (**g**) The superimposition of the TNP-ATP-bound ckP2X7 (orange) and apo, closed pdP2X7 (gray, PDB ID: 5U1L) structures. The RMSD value is 2.18 Å for 787 Cα atoms between trimers. (**h**) The superimposition of the apo, closed pdP2X7 (gray) and A804598-bound pdP2X7 (gold, PDB ID: 5U1V) structures. The RMSD value is 0.45 Å for 949 Cα atoms between trimers.

Supplementary Table 1 The nucleotide sequences of the synthesized ckP2X7 WT and the crystallization construct ckP2X7$_{cryst}$ used in this study.

ckP2X7 WT seq	ATGGAATTCGTGGCTTGGGGCTGGATGAAGGACGTGTTCAACTACGAGTCCCCCAAGCTGATCCGGTTCCC<br>CTCTGTGGGACTCGTGTGCGTGAAGTGGTTCATCTACGGCGTGATCGCCGTGTACATCTGCTACACCCTGA<br>TCGTGCACAAGCGCTACCAGGAAAAAGAGGAACTGACCAGCTCCGTCAGAGTGACCCTGAAGGGCGTGGC<br>CCACGTGGACAGAATTTGGGACGCCGCCGAGTACACCATCCCCACCCAGACCAGAGACAGCTTCTTCGTG<br>ATGACCAACATCATCCGGACCGAGAACCAGATCCAGAAAACCTGCCCCGAGTACCCCACCGCCAAGGCCA<br>TCTGCAGCAGCGATAAGAGCTGCGCCAAGGGCATCGTGGACGTGCACAGCAATGGCGTGCAGACCGGGAA<br>GTGCGTGCACTACAACATCACCCACAAGACCTGCGAGATCAAGGCCTGGTGCCCTGTGCAGGGCGAAGAA<br>AGACCTCCTGTGCCCGCCGTGCTGAGAAGCAGCGAGGACTTCACCGTGTTCATCAAGAACAACATCCACTT<br>CCCCACCTTCAACTACACCGTGCAGAACATCAGCCCTAAGCTGAACACCAGCTGCAAGTTCAACAAAGTG<br>ACCGCCCCACTGTGCCCCATCTTCCGGCTGGGAGACATTCTGCAGGAAGCCAAAGAAAACTTCAGCGAGA<br>TGGCCGTGAAGGGCGGCATCATTGCCATCGAGATTAAGTGGGACTGCGACCTGGACAGCTGGTCCTACTA<br>CTGCAGCCCTGAGTACAGCTTCCGGCGGCTGGACGACAAGACCAGAACCCAGTACCCCGGCTTCAGCATC<br>AGATTCGCCCGGCACTACAAGCTGCCCGACGGCACCGAACAGCGGACCCTGTTTAAGGCCTACGGCATTA<br>GATTCGACGTGCTGGTGTTTGGCATGGGCGGCCAGTTTAAGCTGATCGAGCTGTTCACCTTCATCGGCAGC<br>ACAATCGCCTACTTCGGCCTGGCCGTGACCATCATCGAGATGTGCTTCCACCTGTACAACTGCAGCTCCTG<br>CTGCAAGATCCAAGTGTGCGAGAACGTGATCCGGAAGAAATACGAGACAGTGCTGATGCCCGAACAAGTG<br>ATCCTGGTGTCCTACGTGGACAAGCCCCACATCACACTGATCAAGATGCCCCTGCGGACCAGCCTGCAGAA<br>CGCCGAGGGCAGCATCTTCGAGGACCACCCCGTGAAGTCTTACGACCCCCGGACCTGCTGCAGCCACAAG<br>AGCAACGAGAAACACGGCGCTGCCCAGAGCGAGCTGAGGCCTCTGACACAGAGCAGCAGCTCCACCAACT<br>GCCCCAAGTGGTGCTGCTGCGGCAGATGCCAGGTGGCCCAGAAGCACCACGAGCAGCTGTGCTGCAGAAA<br>GAAAGAGGGCCAGTGCATCACCACCACCTATTGGTTCGCCCAGCTGGTGCTGAGCCGGGACACACTGAAC<br>AAGGCCCTGCTGTACGAGGACCCCTTCCTGGATCTGACCGGCCACAGCAGCAACAGCCAGCTGAGAAGGA<br>TCGCCTACAAGCAGTACATCCATTGGAGATTCGGCAGCTTCGAGCTGGAAGATCGGGCCATCATCCCCAGC<br>TGCTGTCGGCGGCTGATCAGATCCACCTACCCCAAAGAGAACGGCAATTACACCGGCTTCAACCTGGAACT<br>CGAGTAA
ckP2X7$_{cryst}$ seq	ATGTGCGTGAAGTGGTTCATCTACGGCGTGATCGCCGTGTACATCTGCTACACCCTGATCGTGCACAAGCG<br>CTACCAGGAAAAAGAGGAACTGACCAGCTCCGTCAGAGTGACCCTGAAGGGCGTGGCCCACGTGGACAG<br>AATTTGGGACGCCGCCGAGTACACCATCCCCACCCAGACCAGAGACAGCTTCTTCGTGATGACCAACATCA<br>TCCGGACCGAGAACCAGATCCAGAAAACCTGCCCCGAGTACCCCACCGCCAAGGCCATCTGCAGCAGCGA<br>TAAGAGCTGCGCCAAGGGCATCGTGGACGTGCACAGCAATGGCGTGCAGACCGGGAAGTGCGTGCACTAC<br>AACATCACCCACAAGACCTGCGAGATCAAGGCCTGGTGCCCTGTGCAGGGCGAAGAAAGACCTCCTGTGC<br>CCGCCGTGCTGAGAAGCAGCGAGGACTTCACCGTGTTCATCAAGAACAACATCCACTTCCCCACCTTCCAG<br>TACACCGTGCAGAACATCAGCCCTAAGCTGAACACCAGCTGCAAGTTCAACAAAGTGACCGCCCCACTGT<br>GCCCCATCTTCCGGCTGGGAGACATTCTGCAGGAAGCCAAAGAAAACTTCAGCGAGATGGCCGTGAAGGG<br>CGGCATCATTGCCATCGAGATTAAGTGGGACTGCGACCTGGACAGCTGGTCCTACTACTGCAGCCCTGAGT<br>ACAGCTTCCGGCGGCTGGACGACAAGACCAGAACCCAGTACCCCGGCTTCAGCATCAGATTCGCCCGGCA<br>CTACAAGCTGCCCGACGGCACCGAACAGCGGACCCTGTTTAAGGCCTACGGCATTAGATTCGACGTGCTG<br>GTGTTTGGCATGGGCGGCCAGTTTAAGCTGATCGAGCTGTTCACCTTCATCGGCAGCACAATCGCCTACTT<br>CGGCCTGGCCGTGACCATCATCGAGATGTGCTTCCACCTGTACAACCTCGAGTAA

Supplementary Information References

1. Kawate, T., Michel, J. C., Birdsong, W. T. & Gouaux, E. Crystal structure of the ATP-gated P2X(4) ion channel in the closed state. *Nature* **460,** 592–598 (2009).
2. Sluyter, R. & Stokes, L. Significance of P2X7 receptor variants to human health and disease. *Recent Pat. DNA Gene Seq.* **5,** 41–54 (2011).
3. Roger, S. *et al.* Single nucleotide polymorphisms that were identified in affective mood disorders affect ATP-activated P2X7 receptor functions. *J. Psychiatr. Res.* **44,** 347–355 (2010).
